# Synthesis of Dimethyl Carbonate by Transesterification of Propylene Carbonate with Methanol on CeO_2_-La_2_O_3_ Oxides Prepared by the Soft Template Method

**DOI:** 10.3390/ma14174802

**Published:** 2021-08-24

**Authors:** Maria Giorgia Cutrufello, Luciano Atzori, Daniela Meloni, Alessandra Piras, Delia Gazzoli, Elisabetta Rombi

**Affiliations:** 1 Dipartimento di Scienze Chimiche e Geologiche, Università di Cagliari—Complesso Universitario di Monserrato, 09042 Monserrato, Italy; gcutrufe@unica.it (M.G.C.); lucianoatzori@hotmail.it (L.A.); dmeloni@unica.it (D.M.); 2 Consorzio Interuniversitario Nazionale per la Scienza e Tecnologia dei Materiali (INSTM), Unità di Cagliari, 50121 Firenze, Italy; 3 Institute for Materials Research, Hasselt University, 3590 Diepenbeek, Belgium; arla_piras@msn.com; 4 Chemistry Department, Namur University, 5000 Namur, Belgium; 5 Dipartimento di Chimica, Università di Roma “La Sapienza”, 00185 Roma, Italy; delia.gazzoli@uniroma1.it

**Keywords:** CeO_2_-La_2_O_3_ oxides, soft template method, dimethyl carbonate, propylene carbonate transesterification, adsorption microcalorimetry

## Abstract

In this study, CeO_2_, La_2_O_3_, and CeO_2_-La_2_O_3_ mixed oxide catalysts with different Ce/La molar ratios were prepared by the soft template method and characterized by different techniques, including inductively coupled plasma atomic emission spectrometry, X-ray diffraction, N_2_ physisorption, thermogravimetric analysis, and Raman and Fourier transform infrared spectroscopies. NH_3_ and CO_2_ adsorption microcalorimetry was also used for assessing the acid and base surface properties, respectively. The behavior of the oxides as catalysts for the dimethyl carbonate synthesis by the transesterification of propylene carbonate with methanol, at 160 °C under autogenic pressure, was studied in a stainless-steel batch reactor. The activity of the catalysts was found to increase with an increase in the basic sites density. The formation of dimethyl carbonate was favored on medium-strength and weak basic sites, while it underwent decomposition on the strong ones. Several parasitic reactions occurred during the transformation of propylene carbonate, depending on the basic and acidic features of the catalysts. A reaction pathway has been proposed on the basis of the components identified in the reaction mixture.

## 1. Introduction

Dimethyl carbonate (DMC) is an environmentally friendly chemical compound that is widely used in the chemical industry [1]. It has little ecotoxicity and its persistence and bioaccumulation in living organisms are low [2,3]. DMC is widely used as a precursor for polycarbonate resins and as an advantageous carbonylation and methylation agent, replacing the extremely toxic phosgene, dimethyl sulfate, and dimethyl halides in various applications [1,2]. It is used as a substitute for many conventional solvents [4,5,6,7,8] and for producing glycerol carbonate [4,5] and diphenyl carbonate [6]. DMC is also an alternative oxygenate additive in gasoline-based fuels, which reduces the emission of solid particulates and NO*_x_* [1,2,3]; it has a higher oxygen content on a weight basis (53.3%) compared to methanol (50%), ethanol (34.89%), and methyl tert-butyl ether (17.6%), the latter being toxic and poorly biodegradable. In addition, DMC can be used as an electrolyte in lithium batteries [2].

Traditionally, DMC was produced through the phosgenation of methanol, which suffered from toxicity and corrosion problems, as well as from the generation of large amounts of HCl and of inorganic salts as waste products, and was therefore abandoned in the 1980s. Currently, DMC is mainly produced by the methanol oxycarbonylation process [1,2,3]; however, this has some drawbacks, such as the production of significant amounts of CO_2_ and the use of a corrosive Cu halide as a homogeneous catalyst that causes difficulties in product separation. The carbonylation of methyl nitrite on carbon-supported Pd is also used to obtain DMC with high yields, but the cost of the catalyst, the toxicity of NO_x_, and the difficult post-treatment of methyl nitrate are significant problems. One environmentally friendly route for obtaining DMC is the direct synthesis from methanol and CO_2_; however, this is limited by thermodynamic constraints. The transesterification of cyclic (propylene or ethylene) carbonates with methanol is a promising route that does not lead to wastes or the corrosion of equipment. This route requires a relatively simple process and mild reaction conditions, achieving high catalytic performance and high purity of the end product. At the same time, the co-product propylene (or ethylene) glycol is also a high added-value chemical that is widely used in the chemical industry. The transesterification process can be carried out using a one-step or a two-step method: the former, starting from propylene oxide (or ethylene oxide), CO_2_, and methanol, has a variable product distribution and a low DMC selectivity, while the latter is greener and more efficient, thus having the greatest potential for long-term large-scale industrial development [9]. In particular, the synthesis of DMC through the two-step transesterification of propylene carbonate (PC) with methanol has the advantage that propylene oxide (PO) and CO_2_ can almost completely convert to PC under moderate conditions. Therefore, the improvement of PC conversion and DMC selectivity in the transesterification step is the crucial point of this path. Currently, the common industrial catalyst for PC transesterification is the highly active CH_3_ONa [9]; however, this is extremely sensitive to H_2_O and CO_2_, the presence of which in trace amounts would lead to the formation of inactive Na_2_CO_3_ and/or CH_3_OCOONa. CH_3_ONa also gives rise to environmental problems because of the discharge of alkaline wastewater. Therefore, although homogenous catalysts exhibit high activity, their complex production, separation, and recyclability lead to an increase in equipment investment costs and in energy consumption [10]. Instead, heterogeneous catalysts have greater stability and longer life, and can be recycled and disposed of more easily [11]. For this reason, in the last few decades, researchers’ attention has been drawn to the development of new heterogeneous catalysts. A variety of metal oxides were studied as catalysts for the propylene carbonate esterification reaction [9]. Among these, cerium and lanthanum oxides, either pure or as mixed oxides, have only been reported in a few papers [12,13,14,15].

**Scheme 1:**
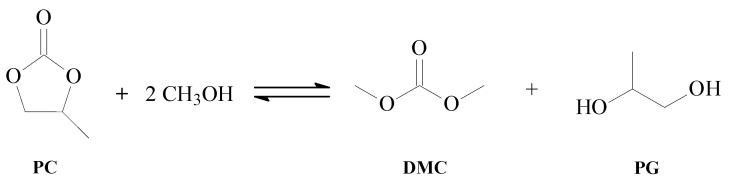
Synthesis of DMC through PC transesterification with methanol.

In the present work, pure ceria and lanthana, as well as CeO_2_-La_2_O_3_ mixed oxides, were synthesized by the soft template (ST) method with different Ce/La molar ratios, characterized by different physicochemical techniques, and used in the transesterification reaction of propylene carbonate with methanol to obtain DMC according to Scheme 1. The effect of the surface’s basic properties on catalytic activity and DMC selectivity was thoroughly investigated.

## 2. Materials and Methods

### 2.1. Materials

Cetyl-trimethyl-ammonium bromine (CTAB, ≥98%), cerium nitrate (Ce(NO_3_)_3_·6H_2_O, 99%), lanthanum nitrate (La(NO_3_)_3_·6H_2_O, 99.9%), sodium hydroxide (NaOH, 98%) methanol (CH_3_OH, 99.8%), nitric acid (HNO_3_, 69 wt.%), propylene carbonate (PC, 99.7%), dimethyl carbonate (DMC, 99%), propylene glycol (PG, 99.5%), and hydrogen peroxide (H_2_O_2_, 35 vol.%) were provided by Aldrich (St. Louis, MI, USA). Ammonia (99.99%) and carbon dioxide (99.95%) were purchased from SIAD (Bergamo, Italy).

### 2.2. Synthesis of Catalysts

Mesoporous ceria, lanthana, and CeO_2_-La_2_O_3_ mixed oxides with different Ce/La molar ratios were synthesized by means of the soft template method [16,17], using CTAB as the templating agent, Ce(NO_3_)_3_·6H_2_O and La(NO_3_)_3_·6H_2_O as precursors, and NaOH as the precipitating agent. For the synthesis, the template and the nitrate precursors were dissolved in suitable amounts (CTAB/precursors: 0.62 mol/mol) in 100 cm^3^ of distilled water under stirring at room temperature. After 30 min, a 0.18 M solution of NaOH was added dropwise until reaching a pH value of 13; after stirring the mixture for 15 h and digestion at 90 °C for 3 h, the resulting solid was separated by filtration and washed with hot water (70 °C). Next, it was dried overnight at 60 °C and then at 110 °C for 6 h, and finally treated in air at 450 °C for 4 h to ensure the complete elimination of CTAB. Thermal decomposition was found to occur between 200 and 350 °C [18], without any aggregation of nanoparticles and consequent decrease in surface areas and pore volumes, which otherwise would occur at temperatures above 450 °C [19]. The synthesized mixed oxides were labeled as CeLa(*x*:*y*), where *x* and *y* indicate the molar percentage of cerium and lanthanum, respectively.

### 2.3. Characterization of Catalysts

Inductively coupled plasma atomic emission spectroscopy (ICP-AES) analyses were performed with a Liberty 200 spectrophotometer (Varian, Palo Alto, CA, USA) to determine La and Ce contents. Samples (ca. 0.015 g) were first dissolved in a mixture of H_2_O_2_ (35 vol.%) and HNO_3_ (69 wt.%) (1:1 by volume) and then diluted with Milli-Q water.

X-ray diffraction (XRD) analyses were performed to investigate the structural properties of the samples by using an ×3000 diffractometer (Seifert, Maitenbeth, Germany) with θ-θ Bragg-Brentano geometry, equipped with a Cu-Kα radiation source and a graphite monochromator before the detector. The Scherrer equation, with the Warren correction, was employed for estimating the average crystallite sizes (*D*_c_) [20].

Textural analysis was carried out with an ASAP 2020 apparatus (Micromeritics, Norcross, GO, USA) by determining the nitrogen adsorption/desorption isotherms at −196 °C. Prior to analysis, the samples were pretreated under vacuum (10^−3^ Pa) at 250 °C for 12 h. The BET method was used for determining the specific surface area (*S*_BET_). The total pore volume (*V*_p_) and the pore size distribution curve (BJH method, isotherm desorption branch) were also assessed.

Thermogravimetric (TG) analyses of the catalysts were carried out on a STA6000 thermal analyzer (PerkinElmer, Waltham, MA, USA). The samples were placed in an alumina crucible and heated under oxygen flow (40 cm^3^ min^−1^) from 30 to 950 °C at a heating rate of 10 °C min^−1^.

Raman spectra were collected at ambient temperature in back-scattering geometry using an inVia micro-Raman spectrometer (Renishaw, Wotton-under-Edge, England, UK) equipped with an air-cooled CCD detector and edge filters. A 514.0 nm emission line from an Ar ion laser was focused on the sample under a Leica DLML microscope, using 20× or 5× objectives. The incident beam had a power of about 5 mW. For each sample, repeated (10 or 20 s) accumulations were normally acquired. The resolution was 2 cm^−1^, and spectra were calibrated using the 520.5 cm^−1^ line of a silicon wafer. Spectra processing included baseline removal and curve fitting using a Gauss–Lorentz cross-product function by Peakfit 4.12 software (Peakfit 4.12, Systat Software Inc., San Jose, CA, USA, 2007, AISN Software).

FTIR characterization was performed on an Equinox 55 spectrometer (Bruker, Ettlingen, Germany), equipped with an MCT cryodetector working at 2 cm^−1^ resolution. Powdered samples were pressed into thin gold self-supporting wafers and then placed into a quartz cell. Spectra were collected at room temperature after outgassing at 450 °C (residual pressure <10^−1^ Pa), exposure to CO_2_ (equilibrium pressure equal to 4 × 10^3^ Pa), and subsequent outgassing for 30 min at room temperature and at 450 °C.

A Tian–Calvet heat flow calorimeter (Setaram, Caluire, France) equipped with a volumetric vacuum line was used for microcalorimetric measurements. Samples (ca. 0.1 g), previously calcined at 450 °C for 4 h, were pre-treated under vacuum (5 × 10^−3^ Pa) at 250 °C for 12 h (heating rate, 1 °C min^−1^). In order to limit non-specific adsorption, the microcalorimetric analyses were carried out at 80 °C. Subsequent doses of the probe gas (ammonia or carbon dioxide) were admitted, and the equilibrium pressure relative to each adsorbed amount was measured by means of a differential pressure gauge, simultaneously recording the thermal effect. The run was stopped at a final equilibrium pressure of 133.3 Pa.

### 2.4. Catalytic Runs

The PC transesterification reaction was carried out in a 100 cm^3^ Teflon-lined stainless steel batch reactor (Parr, Moline, IL, USA), equipped with inlet and exhaust valves for N_2_, a liquid sampling valve, pressure gauge, internal thermocouple, and stirrer. The reactor was located inside a heating mantle to achieve a uniform temperature throughout its volume. The catalysts were tested with a methanol/propylene carbonate ratio of 10 mol/mol and 3 wt.% of catalyst (referred to the propylene carbonate mass).

In a typical run, 7.78 × 10^−2^ mol of propylene carbonate and 7.78 × 10^−1^ mol of methanol CH_3_OH/PC = 10 mol/mol) were charged into the reactor and contacted with 0.24 g of freshly calcined (4 h at 450 °C) catalyst. Thereafter, the reactor was pressurized with nitrogen up to 1.2 bar and heated up to the desired temperature (160 °C) under stirring and autogenic pressure (15 bar). A stirring speed of 300× *g* rpm was used in order to avoid the spreading of the reaction mixture onto the walls of the reaction vessel. After 4 h of reaction, the reactor was cooled down to 5 °C by immersion in an ice bath; the liquid sample was then collected in a closed vial and centrifuged to remove the catalyst from the reaction mixture before analysis. A GC 6890 (Agilent, Santa Clara, CA, USA), equipped with an on-column injector, a Rxi-624Sil MS capillary column (30 m length, 0.25 mm ID, 1.4 μm film thickness) and a flame ionization detector (FID), was used to quantitatively determine the unreacted propylene carbonate and the main reaction products (i.e., dimethyl carbonate (DMC), propylene glycol (PG), 2-hydroxypropyl methyl carbonate (2-HPMC), and 1-hydroxypropan-2-yl methyl carbonate (1-HPMC)) using n-heptane as the internal standard. Besides the major components, other by-products, i.e., dimethyl ether (DME), propylene oxide (PO), 1-methoxy-2-propanol (1MO-2P), 2-methoxy-1-propanol (2MO-1P), and dipropylene glycol isomers (DPGs) were also quantified (Table S1).

The PC conversion (*X*_PC_), DMC selectivity (*S*_DMC_), DMC yield (*Y*_DMC_), and activity (*A*_c_) were calculated according to the following equations:

(1) $$X_{\mathrm{PC}}\;(\mathrm{mol}\;\%)=\frac{n_{\mathrm{PC}}^{\mathrm{reacted}}}{n_{\mathrm{PC}}^{\mathrm{initial}}}\times 100,$$

(2) $$S_{\mathrm{DMC}}\;(\mathrm{mol}\;\%)=\frac{n_{\mathrm{DMC}}^{\mathrm{formed}}}{n_{\mathrm{PC}}^{\mathrm{reacted}}}\times 100,$$

(3) $$Y_{\mathrm{DMC}}\;(\mathrm{mol}\;\%)=\frac{n_{\mathrm{DMC}}^{\mathrm{formed}}}{n_{\mathrm{PC}}^{\mathrm{initial}}}\times 100,$$

(4) $$A_{c}{\;(\mathrm{mol}\;m}^{-2}{\;h}^{-1})=\frac{n_{PC}^{\mathrm{reacted}}/t}{m_{cat}\cdot S_{BET}}\times 100,$$

where *n*_i_ is the number of moles of the *i*^th^ species, $n_{\mathrm{PC}}^{\mathrm{reacted}}\;{=n}_{\mathrm{PC}}^{\mathrm{initial}}-{\;n}_{\mathrm{PC}}^{\mathrm{final}}$, and *m*_cat_ is the mass of the catalyst.

## 3. Results and Discussion

### 3.1. Characterization

The chemical composition of the prepared ceria, lanthana and CeLa mixed oxides is reported in Table 1, where their structural and textural properties are also summarized. Concerning the mixed oxides, the ICP-AES results show that the experimental values of the Ce/La molar ratio are higher than the nominal ones, indicating that the complete incorporation of the La_2_O_3_ oxide into the final solid was not achieved.

**Figure 1:**
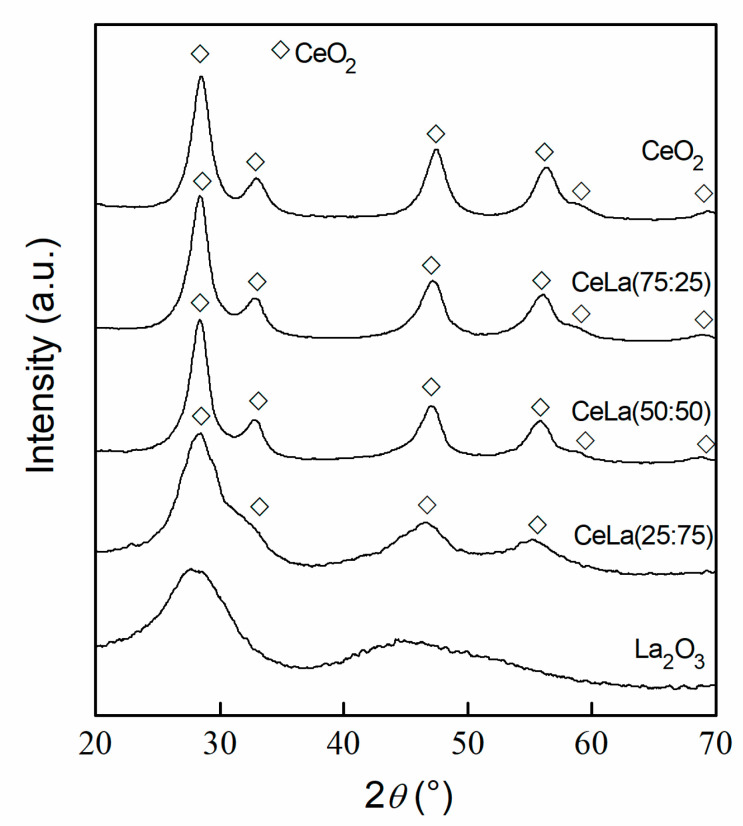
XRD patterns of the prepared oxide catalysts.

The X-ray diffraction patterns of the pure and mixed oxide samples are reported in Figure 1. Reflections at 2*θ* values of 28.5°, 33.1°, 47.5°, and 56.3°, typical for the fluorite-type cubic crystalline structure (PDF card 81-0792), are visible in the XRD pattern of CeO_2_, for which an average nanocrystallite size of 5 nm was calculated. In the diffractogram of La_2_O_3_, two broad signals are visible, which can be ascribed to the formation of an amorphous phase of lanthanum oxide (PDF card 83-1350). Concerning the CeLa mixed oxides, in the XRD pattern of CeLa(75:25) and CeLa(50:50), only the distinctive peaks of the CeO_2_ phase appear, for which an average diameter of the nanoparticles similar to that of the pure oxide was calculated. Conversely, the diffractogram of the CeLa(25:75) sample seems characterized by the dominant contribution of the La_2_O_3_ amorphous phase, which overlaps with the reflections of ceria, not allowing an assessment of the average nanocrystallite size. The presence of La_2_O_3_ as an amorphous phase in CeLa mixed oxides was already observed in the literature for samples prepared via sol-gel [21] and co-precipitation [14].

**Figure 2:**
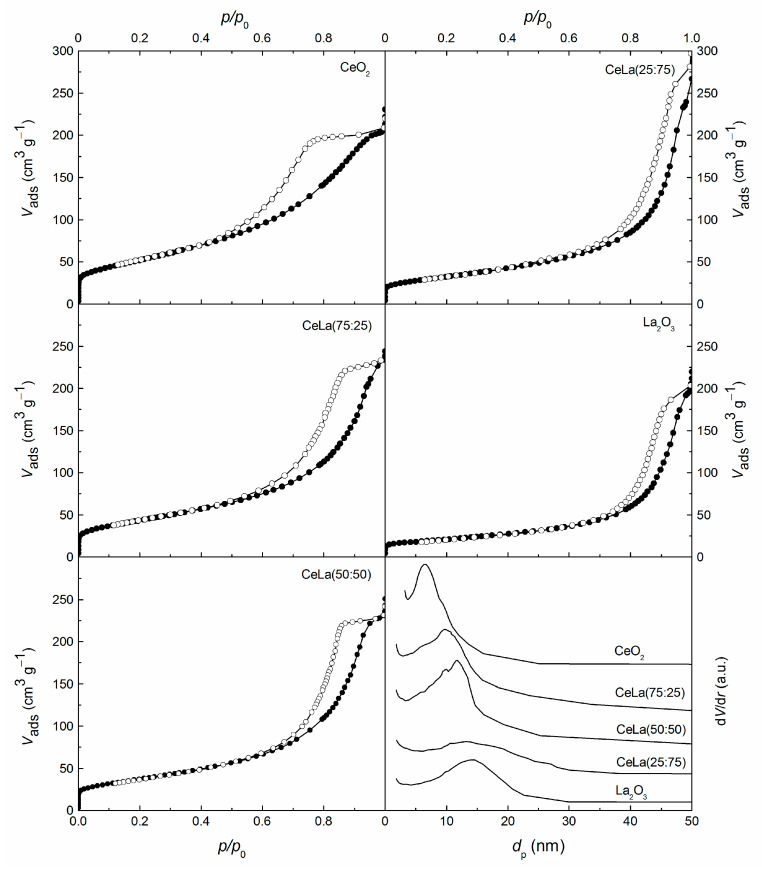
N_2_ physisorption isotherms and pore size distribution plots of the prepared oxide catalysts.

The nitrogen adsorption-desorption isotherms of all the samples (Figure 2) can be classified as type IVa [22] and exhibit hysteresis loops at a relative pressure >0.4, which suggests the presence of mesopores in a limited size range. With the increase in the lanthanum content, the surface areas (*S*_BET_) strongly decrease from 189 to 74 m^2^ g^−1^, whereas pore volumes (*V*_p_) are in the range of 0.31–0.37 cm^3^ g^−1^. As can be observed, the *S*_BET_ values of the present samples are definitely high in comparison with those reported in the literature for CeO_2_-La_2_O_3_ mixed oxides prepared by coprecipitation (41–62 m^2^ g^−1^) [14], highlighting the effectiveness of the soft template method for the synthesis of materials with high surface areas. The pore size distribution curves (PSD, inset in Figure 2) indicate a monomodal distribution, where the maximum (*d*_p,max_) shifts to larger values as the lanthanum content increases (Table 1).

**Figure 3:**
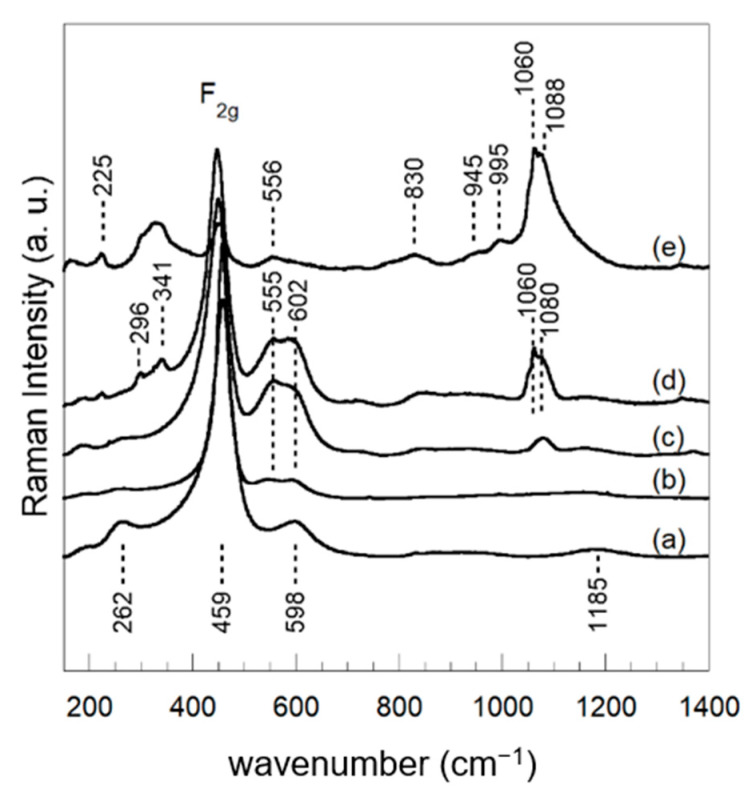
Raman spectra of the prepared oxide catalysts, freshly calcined in air at 450 °C for 4 h: (a) CeO_2_; (b) CeLa(75:25); (c) CeLa(50:50); (d) CeLa(25:75); (e) La_2_O_3_.

The results of the Raman characterization for CeO_2_, La_2_O_3_, and the CeLa(*x*:*y*) mixed oxides, freshly calcined at 450 °C for 4 h, are shown in Figure 3.

Apart from a strong peak at about 459 cm^−1^, ascribed to the F_2g_ mode of the fluorite-like phase, the spectrum of the CeO_2_ sample (Figure 3, curve (a)) exhibits weak bands at about 262, 598, and 1185 cm^−1^, due to second-order transverse acoustic (2TA), defect-induced (D) mode, and second-order longitudinal optical (2LO) mode, respectively [23,24,25]. The F_2g_ mode peak is expected to shift to a lower frequency, and to broaden with a low-frequency tail, with decreasing particle size [26].

In the Raman spectra of the CeO_2_-La_2_O_3_ mixed oxides (Figure 3, curves (b)–(d)), the F_2g_ mode peak shifts to lower frequencies and broadens with a low-frequency tail at increasing lanthanum doping, with a parallel increase in the intensity and complexity of the defect-induced (D) mode. The shift of the F_2g_ mode indicates a strong interaction between the CeO_2_ and La_2_O_3_ species, with a consequent weakening of the Ce–O bond. At increasing lanthanum content, the defect-induced (D) band at about 598 cm^−1^ splits into two components, indicating the occurrence of a solid solution formation [27]. The contributions at about 555 cm^−1^ and at about 602 cm^−1^ are attributed to oxygen vacancies and to cation substitution in the lattice, respectively [28,29]. In the spectrum of CeLa(50:50) (Figure 3, curve (c)) and more markedly in the one of CeLa(25:75), which has the highest La content (Figure 3, curve (d)), additional bands appear at about 296, 341, 1060, and 1080 cm^−1^. These bands suggest the presence of surface lanthanum-containing species that can be reasonably attributed to lanthanum carbonates [30].

In the literature, various proposals for the Raman spectra of La_2_O_3_ can be found, depending on the structure, morphology, and environment. La_2_O_3_ (A-type) is predicted to have four characteristic bands (2E_g_) + (2A_1g_) and two acoustic modes [31,32]. Bands at about 107 cm^−1^ (E_g_), 195 cm^−1^ (A_1g_) and 408 cm^−1^ have been reported for pure La_2_O_3_ [31,32,33], whereas other studies identified an intense Raman band at 405 cm^−1^ [34] and bands at 27, 283, 341, and 451 cm^−1^ [35]. Exposure of La_2_O_3_ to air leads to the rapid formation of La(OH)_3_ (bands at about 138, 227, 281, 339, 449, 605, and 649 cm^−1^), LaOOH (bands at about 135, 202, 216, 312, 344, 383, and 424 cm^−1^) [36], and to carbonation processes. Compounds corresponding to I-La_2_O_2_CO_3_ (bands at about 670, 712, 868 and 1064 cm^−1^) [36], Ia-La_2_O_2_CO_3_ (bands at about 290, 333, 438, 451, 1052, and 1341 cm^−1^) and/or to II-La_2_O_2_CO_3_ (bands at about 355, 385, 740, and 1082 cm^−1^) have been recognized [30,36]. The Raman profile recorded on La_2_O_3_ (Figure 3, curve e) shows several low-intensity bands in the range of 200–900 cm^−1^ and a strong complex band at about 1075 cm^−1^. Although it is difficult to assign each band to a specific compound, the strong band at 1075 cm^−1^, with components at about 1060 and 1080 cm^−1^, identifies the presence of La-carbonate species (ν_1_ symmetric stretching).

In addition to Raman spectroscopy, the FTIR technique provides a powerful tool for investigating the surface properties of solid catalysts, by using suitable probe molecules to have information on the adsorbent-adsorbate interactions. In particular, the nature and strength of basic hydroxyl groups (OH) and basic surface oxygens (O^2−^) can be studied through their reaction with CO_2_, which produces, respectively, hydrogen carbonate and carbonates species [37,38,39,40,41]. Therefore, FTIR analyses were performed on the CeO_2_, CeLa(50:50), and La_2_O_3_ samples after outgassing at 450 °C, exposure to CO_2_ (equilibrium pressure equal to 4 × 10^3^ Pa) at room temperature (RT), and subsequent outgassing at RT and at 450 °C.

**Figure 4:**
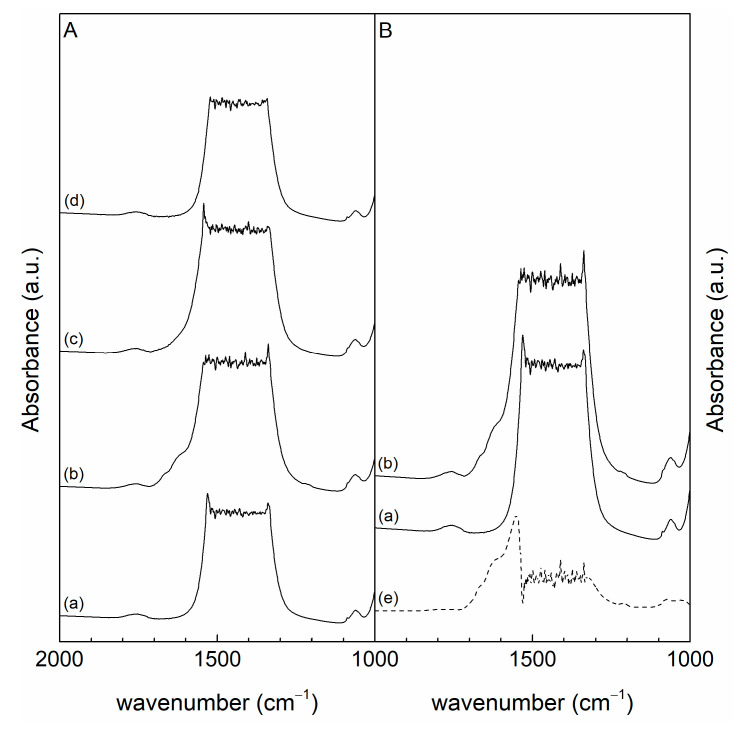
FTIR spectra at RT after outgassing at 450 °C (a), exposure to CO_2_ (b), and subsequent outgassing at RT (c) and 450 °C (d).

The FTIR spectra of CeO_2_ in the region of 2000–1000 cm^−1^ are shown in Figure 4. Compared to the spectrum collected after the first treatment at 450 °C, CO_2_ adsorption leads to the appearance of different bands. In the range of 1700–1200 cm^−1^, a broad signal, clearly consisting of distinct bands, highlights the presence of a heterogeneous surface, on which basic sites of different natures and strengths coexist [37,39]. In particular, at least five partially superimposed contributions can be observed, due to the vibrational modes of (CO_3_) asymmetrical/symmetrical stretching (ν_as(CO__3)_ and ν_s(CO__3)_, respectively) and of (OH) bending (δ_(OH)_), characteristic of hydrogen carbonates and different carbonate species. According to the literature [39], the most intense band, centered at ca. 1594 cm^−1^, can probably be ascribed to the overlap of two contributions, i.e., the ν_as(CO__3)_ modes of hydrogen carbonates (HC) and bidentate carbonates (BC), typically observed at around 1600 and 1570 cm^–1^, respectively. Indeed, the presence of these species is also clearly indicated by the presence of the HC bands at 1406 and 1217 cm^−1^ (ν_s(CO__3)_ and δ_(OH)_, respectively), and by the signal at 1298 cm^−1^ (ν_s(CO__3)_) ascribable to BC. In addition, the presence of monodentate carbonates (MC) is also suggested by the presence of a little hump at ca. 1500 cm^−1^ (ν_as(CO__3)_), while the tail at higher frequencies of the band at 1406 cm^−1^ could be considered to be an indication of the existence of polydentate and bridged carbonates (whose signals are typically observed at 1465 [39] and 1430 cm^−1^ [41], respectively). Outgassing at RT leads to a general lowering of the bands’ height, also accompanied by significant changes in their relative intensities. The low stability of HC is indicated by the almost-disappearance of their bands; therefore, a previously hidden signal centered at 1685 cm^−1^ emerges, reasonably ascribable to bridged carbonates [41]. The ν_as(CO__3)_ contribution centered at 1565 cm^−1^, attributable to bidentate carbonates, now appears more evident, underlining the higher stability of BC with respect to HC. However, basic O^2−^ sites generating bidentate carbonates turn out to be weaker than those on which monodentate carbonates are formed. Indeed, after outgassing at RT, the ν_s(CO__3)_ stretching mode of MC, centered at 1500 cm^−1^, appears as the most intense band of the spectrum. At lower frequencies, a wide signal located at ca. 1336 cm^−1^ can still be observed, probably due to different contributions, such as the vibrational modes of polydentate carbonates (ν_as(CO__3)_), monodentate carbonates (ν_s(CO__3)_), and bridged carbonates (typically observed at 1390, 1350, and 1320 cm^−1^, respectively), as well as those of residual bidentate carbonates. When outgassing is performed at 450 °C, the pertinent spectrum clearly shows a further decrease in overall signal intensity, for which, as expected, the most important contributions are related to the presence of highly thermally stable polydentate carbonates [38,39].

As for the CeLa(50:50) catalyst, the spectrum collected after the first treatment at 450 °C (Figure 4) shows the presence of two intense bands centered at ca. 1465 cm^−1^ and 1392 cm^−1^, mainly ascribable to polydentate carbonates, i.e., bulk-like or subsurface carbonates. These highly stable species are most probably formed by the interaction between atmospheric carbon dioxide and the strongest basic sites on the catalyst surface. It is worth noting that much less significant bands of such polydentate carbonates were observed in the analogous spectrum of pure ceria, indicating the superior basic character of the CeLa(50:50) sample and confirming what was indicated by the Raman results. Additionally, in this case, CO_2_ adsorption causes the growth of the bands due to the formation of hydrogen carbonates and carbonate species. At least four bands can be individuated in the range of 1700–1200 cm^−1^, not all of which are uniquely ascribable to specific species. An important increase in the contributions of HC and BC species is clearly underlined by the appearance of the signal centered at about 1605 cm^−1^, due to the superimposition of the corresponding ν_as(CO__3)_ vibrational modes. The presence of the hydrogen carbonates is also clearly indicated by the minor band at 1214 cm^−1^. Intriguingly, the hump whose maximum is located at ca. 1500 cm^−1^ seems to suggest a prominent contribution of monodentate carbonates, probably greater than those of HC and BC; this feature appears much more significant than that observed in the corresponding spectrum of CeO_2_, highlighting further the existence of a greater percentage of stronger basic sites in the presence of lanthanum. Outgassing at RT leads to the smoothing of the overall signal at higher frequencies, suggesting the partial desorption of the HC and BC species. After further outgassing at 450 °C, the thermally less stable species desorb from the catalyst surface, restoring the spectrum observed before CO_2_ adsorption.

The marked tendency of lanthana to react with atmospheric carbon dioxide to form very stable bulk-like carbonates, especially in the presence of humidity, was reported by several authors, as revealed by FTIR [42,43] and Raman results [36]. Accordingly, the spectrum of La_2_O_3_ after the first thermal treatment at 450 °C (Figure S1A) shows some very intense signals (the absorbance value goes to saturation) related to the formation of the more stable carbonate species, i.e., polydentate carbonates and probably some monodentate carbonate species. Such a feature is much more evident than for CeO_2_ and CeLa(50:50), confirming a rise in the concentration of very strong surface basic sites by increasing the lanthana content. Nevertheless, a pure La_2_O_3_ surface cannot be considered completely saturated by such remarkably stable carbonates since the CO_2_ adsorption leads to the appearance of additional bands, as highlighted by the difference spectrum (Figure S1B), obtained by subtracting the IR profile acquired after the first thermal treatment from that collected after CO_2_ adsorption. Finally, outgassing at RT and 450 °C progressively restores the initial condition.

The thermogravimetric curves of the prepared catalysts are reported in Figure S2, and the calculated weight losses in different temperature ranges are summarized in Table S2. The pure cerium oxide shows a weight loss of about 6 wt.% below 200 °C, which can be attributed to the elimination of physically adsorbed water (Figure S2A). No significant weight losses are observed at higher temperatures, confirming the absence of the residual template after calcination at 450 °C. Conversely, in the case of pure La_2_O_3_, remarkable weight losses occur in all the investigated temperature regions: besides the contribution in the low-temperature range, peaks centered at about 250, 400, and 750 °C are visible in its DTG curve (Figure S2B), which indicate that different thermal processes are taking place. According to the literature [21,44], such losses can be ascribed to the presence of carbonate-like species that easily form on the La_2_O_3_ surface and decompose at different temperatures because of their different stabilities. The thermogravimetric curves of the CeLa(*x*:*y*) mixed oxides also show various weight losses above 200 °C (Figure S2A,B), which can be ascribed to the decomposition of the carbonate species formed on lanthana. Although for CeLa(25:75) the number of carbonate species that decompose in the range 200–400 °C is lower in comparison with the other CeO_2_-La_2_O_3_ mixed oxides, the amount of carbonate species decomposing at a higher temperature is greater. These findings are in agreement with those obtained from Raman and FTIR characterization, confirming the formation of stable carbonate-like species on the La_2_O_3_ surface for the catalysts with La/Ce molar ratios ≥1.

**Figure 5:**
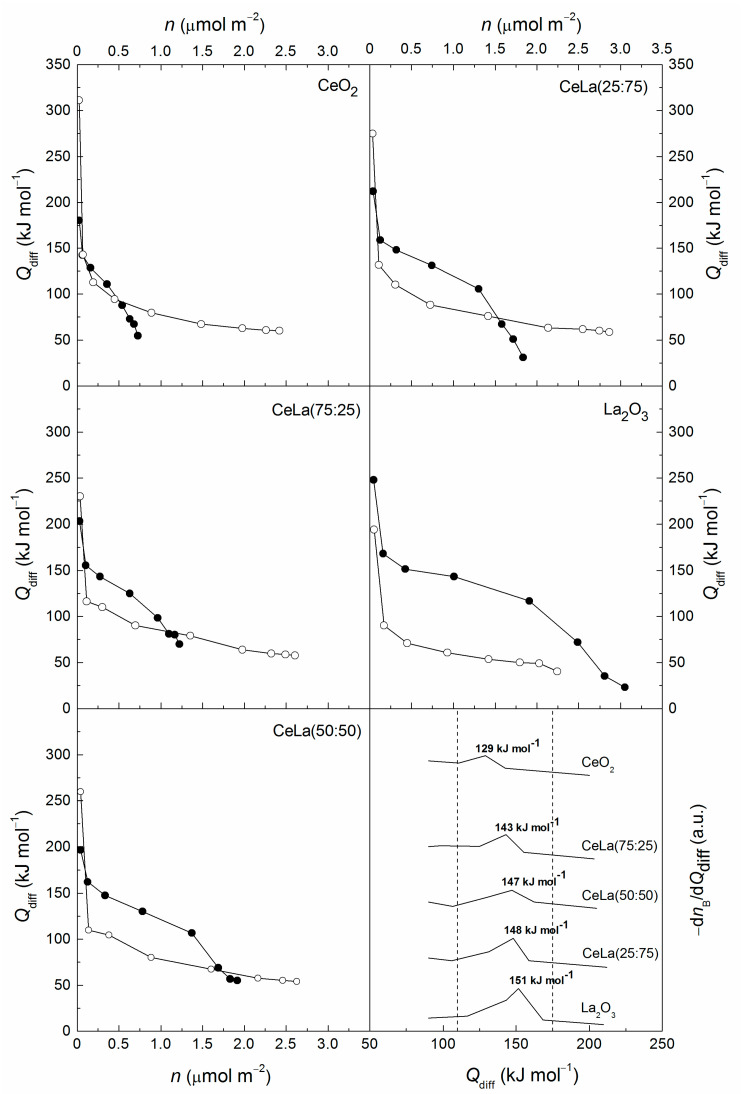
Differential heats of adsorption vs. probe molecule uptake and basic site energy distribution plots: (●) carbon dioxide; (○) ammonia.

The calorimetric results of NH_3_ and CO_2_ adsorption for all the samples are summarized in Table 2 and Figure 5, where the differential heat of adsorption, *Q*_diff_, is reported as a function of the amount (*n*, μmol m^−2^) of the adsorbed probe molecule.

Concerning NH_3_ adsorption, high initial *Q*_diff_ values are observed, which reveal the presence of strong acid sites. Such values decrease from 311 to 194 kJ mol^−1^ with the increase in the La content. For all the samples, the differential heat decreases as the ammonia amount adsorbed increases, thus indicating the heterogeneity of the acid sites. Surface site heterogeneity is expected, due to the presence of cations with different chemical environments acting as Lewis acid sites, as well as to the presence of Brønsted acidic OH groups originating from the occurrence to some extent of surface hydroxylation [45,46]. The contribution of physisorption, which can take place at high uptake values, should be neglected in the assessment of the acid site (*n*_A_) concentration. Differential heats that are as high as twice or three times the condensation heat of the probe molecule (20.2 kJ mol^−1^ at 80 °C for ammonia) are generally considered to be the threshold value between chemical and physical or non-specific adsorption. Accordingly, the fraction of ammonia uptake corresponding to differential heats below 60 kJ mol^−1^ was neglected when assessing the site strength distribution (Table 2) by roughly ranking the acid sites as strong (*n*_A,s_, *Q*_diff_ ≥ 150 kJ mol^−1^), medium-strength (*n*_A,m_, 110 ≤ *Q*_diff_ < 150 kJ mol^−1^), and weak (*n*_A,w_, 60 ≤ *Q*_diff_ < 110 kJ mol^−1^). It can be observed that all the samples show a similar concentration of strong acid sites, whereas the amount of medium-strength and weak acid sites of ceria and CeO_2_-La_2_O_3_ mixed oxides is one order of magnitude higher than that of La_2_O_3_.

Concerning basicity, the curves of differential heat for CO_2_ adsorption reveal the presence of strong basic sites on all the samples, as indicated by the high initial values of *Q*_diff_, which increase from 181 to 248 kJ mol^−1^ with the increase in La loading. A clear correlation between the lanthanum content and the increasing concentration of basic sites (*n*_B_), calculated from the total CO_2_ uptake by disregarding the portion corresponding to differential heats lower than 40 kJ mol^−1^ (i.e., about three times the condensation heat of CO_2_ at 80 °C, 13.6 kJ mol^−1^), can be observed (Table 2). Furthermore, for pure lanthanum oxide, the ratio between the total amount of basic and acid sites (*n*_B,tot_/*n*_A,tot_) is more than 3 times higher than for the other samples, pointing out its definitely predominant basic character.

From the *Q*_diff_ vs. carbon dioxide uptake profiles, the site-energy distribution plots can be obtained, where the negative inverse of the first derivative of the differential heat with respect to *n*_B_ (–d*n*_B_/d*Q*_diff_) is plotted vs. *Q*_diff_. The presence of peaks in such plots reveals that sets of energetically homogenous sites are present on the surface: the higher the area under the peak, the higher the population of the corresponding family of sites. In the present case, in the site-energy distribution plots of all the samples, a single peak is visible in the region of *Q*_diff_ that is typical for chemical adsorption (Figure 5), which indicates the presence of a family of sites with the same adsorption energy, whose amount and strength increase as the lanthanum content increases, as indicated by the larger peak area and the increasing *Q*_diff_ values (from 129 to 151 kJ mol^−1^) going from pure ceria to pure lanthana.

### 3.2. Catalytic Runs

Catalytic results for the transesterification of PC with methanol at 160 °C under autogenic pressure are reported in Table 3. The reaction was carried out using a CH_3_OH/PC molar ratio equal to 10, which was found to be the optimum for the synthesis of DMC on different catalysts, such as ionic liquids, hydrotalcites, and metal oxides [9]. The use of high methanol/PC molar ratios is advantageous as it shifts the equilibrium toward the right side, due to the formation of a DMC–methanol azeotrope as a consequence of the excess of methanol [14,47].

In order to evaluate the influence of the thermal reaction, a blank run was also performed, which accounts for about 15 mol% of PC conversion and 11 mol% of DMC selectivity, the most abundant products being 1- and 2-hydroxypropan-2-yl methyl carbonate intermediates (46 mol%) (Table 3). The total selectivity far from 100 mol% indicates that some side reactions take place to a considerable extent in the absence of the catalyst, as also suggested by the DMC/PG ratio (0.46), which has a value considerably lower than the expected value based on the 1:1 stoichiometry of formation of the DMC and PG co-products. Only a few articles concerning the catalyst-free transesterification of cyclic carbonates can be found in the literature [19,48,49]. No thermal conversion was observed during the propylene carbonate transesterification with methanol at 130 °C [48]. Conversely, in the case of the more reactive ethylene carbonate (EC), a conversion of 18 mol% and a selectivity to the 2-hydroxyethyl methyl carbonate intermediate of ca. 54 mol% were determined after 2 h at 140 °C for the thermal reaction of EC on mesoporous ceria catalysts [19].

**Figure 6:**
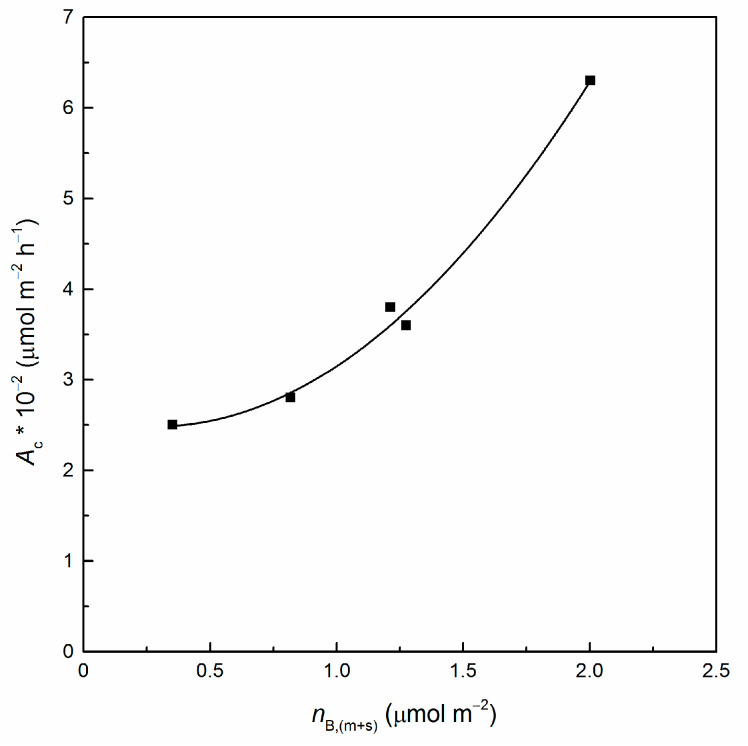
Catalytic activity as a function of medium-strength and strong basic sites’ concentration. Experimental conditions: *T* = 160 °C; autogenic pressure (15 bar); CH_3_OH/PC = 10 mol/mol; catalyst/PC = 0.03 g/g; reaction time = 4 h.

Regardless of the oxide used, the reaction in the presence of the catalyst leads to a great increase in both the PC conversion (55–60 mol%) and DMC selectivity (74–87 mol%). Unlike the conversion, for which there is no clear correlation with the lanthanum content, the catalytic activity (*A*_c_) increases from 0.25 to 0.63 mmol m^−2^ h^−1^ as the lanthanum content increases (Table 3). This trend can be explained by considering the increase in the concentration of the surface basic sites at decreasing Ce/La molar ratios, as proved by the results of the CO_2_ adsorption microcalorimetry (cf. Table 2). As shown in Figure 6, a very good correlation exists between the concentration of medium-strength and strong basic sites, *n*_B,(m+s)_ (i.e., the CO_2_-adsorbing sites with *Q*_diff_ ≥ 110 kJ mol^−1^) and the activity, which increases markedly as *n*_B,(m+s)_ increases, highlighting the fundamental role of the basic sites’ density and strength in the PC transformation on the investigated catalysts.

Conversely, no correlation is evident between catalytic activity and acidity. These results are in agreement with most of the literature on DMC synthesis from PC and methanol, where the activity was ascribed to the high basicity of the catalysts in terms of both the concentration and strength of the sites, while no relevant involvement of the acid sites was found [9,19,47,50,51,52,53,54,55]. In particular, the addition of lanthanum was reported to be beneficial for the catalytic activity of a series of CeO_2_-La_2_O_3_ catalysts prepared by co-precipitation, which was ascribed to the increase in the amount and strength of the surface basic sites, along with the La content [14]. Only a few authors considered the concurrent participation of acid and basic sites as responsible for an enhancement in catalytic activity [14,51].

**Figure 7:**
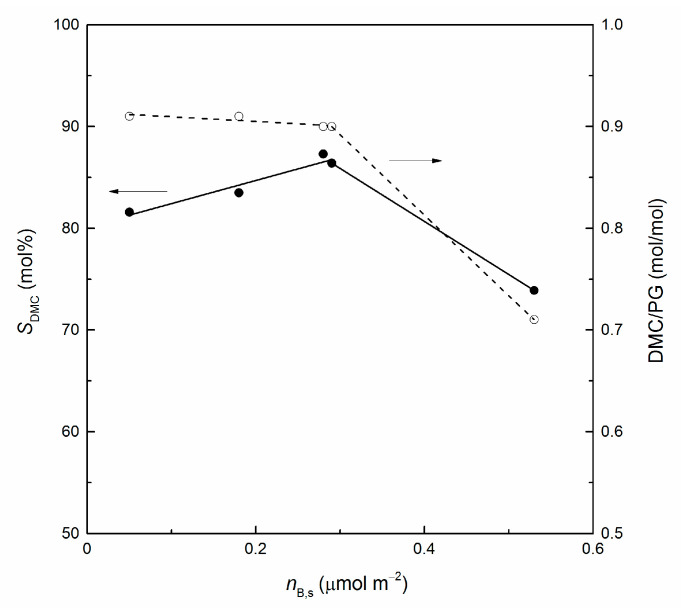
DMC selectivity (●) and DMC/PG molar ratio (○) as a function of the strong basic sites’ concentration. Experimental conditions: *T* = 160 °C; autogenic pressure (15 bar); CH_3_OH/PC = 10 mol/mol; catalyst/PC = 0.03 g/g; reaction time = 4 h.

Concerning the selectivity to DMC, an increase takes place from 82 to 87 mol% going from CeO_2_ to CeLa(25:75), whereas a marked reduction to 74 mol% occurs in the case of La_2_O_3_ (Table 3). It is worthy of note that CeLa(50:50) and CeLa(25:75), which have a comparable concentration of medium-strength and strong basic sites, also show similar values of both catalytic activity and DMC selectivity. Moreover, a DMC/PG molar ratio of ca. 0.9 is observed for all catalysts except lanthana, for which it decreases to 0.71. These values, below the stoichiometric one, still suggest the occurrence of side reactions, in particular on the pure lanthanum oxide, although this is to a minor extent compared to the thermal reaction. Both DMC selectivity and the DMC/PG molar ratio clearly depend on the concentration of strong basic sites (*n*_B,s_, Q_diff_ > 150 kJ/mol), as shown in Figure 7. While the DMC/PG molar ratio remains quite constant, S_DMC_ linearly increases up to *n*_B,s_ values of 0.3 μmol m^−2^, but both parameters abruptly decrease for the highest number of strong sites, which corresponds to lanthana. DMC was found to easily hydrolyze to methanol and CO_2_ on the strong basic sites of a commercial MgO below 100 °C, and also to decompose to DME and CO_2_ on very strong basic sites beyond this temperature [56]. For the present catalysts, it is worth considering that, as the lanthanum content increases, the strong basic sites not only increase in terms of concentration but also in terms of strength, as indicated by the shift of the maximum of the site-energy distribution plots to higher values of *Q*_diff_ (cf. Figure 5). The observed trend of DMC selectivity as a function of basicity can be explained according to what is reported in the literature on DMC decomposition [56], the formation of CO_2_ being qualitatively indicated by the presence of numerous bubbles in the reaction mixture.

Regarding the DMC/PG molar ratio, it must be considered that this value is affected at the same time by the amounts of DMC and PG. By looking at Table S1 (which reports the molar percentage of the products detected by GC analysis), it can be observed that, contrary to what happens for DMC, the quantity of PG is significantly higher for lanthana. This suggests that on this catalyst, PG is also formed through reactions other than PC transesterification, such as the decomposition of PC to CO_2_ and PG in the presence of H_2_O [11,57,58], the latter being possibly formed by methanol dehydration on weak and medium-strength acid sites [59]. In addition, the presence of dipropylene glycols (DPGs), although in low amounts, indicates that PG may also be involved in etherification reactions [57,60].

Concerning PC transformation, besides the above-mentioned reactions, the decomposition of PC to PO and CO_2_ can also occur [61], which may be followed by the methanolysis of PO to 1-methoxy-2-propanol (1MO-2P), mainly on basic sites, and 2-methoxy-1-propanol (2MO-1P), mainly on acid sites [12,62]. For the present catalysts, the formation of the methoxy-propanol isomers (Table S1) indicates that methanolysis of propylene oxide actually takes place. The amounts of both the 1MO-2P and 2MO-1P isomers are found to decrease in the presence of lanthanum. On La_2_O_3,_ which possesses the highest concentration of strong basic sites as well as the lowest acidity, no formation of 1MO-2P is observed. This result, which is at first sight surprising, can be explained according to the literature [62], where catalysts with different acid-base properties (i.e., MgO, CaO, and Al_2_O_3_) were used. It was reported that only basic sites with moderate strength were able to dissociate methanol to methoxide ions and to abstract a proton from propylene oxide to produce 1-methoxy-2-propanol; conversely, strong basic sites did not favor 1MO-2P formation because of the strong adsorption of methanol, whereas weak basic sites were not active at all for the reaction between CH_3_OH and PO.

**Scheme 2:**
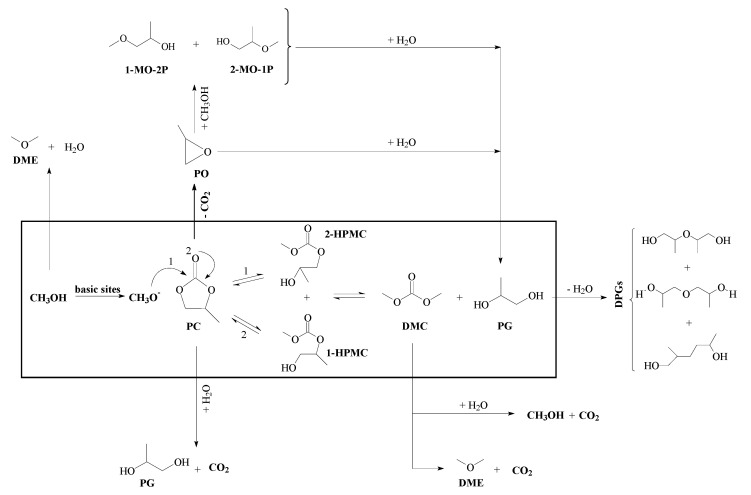
Reaction pathway for propylene carbonate transesterification with methanol on the prepared oxide catalysts.

On the basis of the catalytic results discussed above, a plausible reaction pathway is proposed in Scheme 2 where, in addition to the main reaction of DMC formation, the side reactions assumed on the basis of the components identified in the reaction mixture are also presented.

## 4. Conclusions

In the present work, pure ceria and lanthana, as well as CeO_2_-La_2_O_3_ mixed oxides, were synthesized by the soft template method with different Ce/La molar ratios. They were proved to be active in the reaction between propylene carbonate and methanol to obtain DMC. The surface acid and basic properties were investigated by the adsorption microcalorimetry of NH_3_ and CO_2_, respectively. It was found that the basic character of the catalysts increases both in terms of concentration and strength of the sites with an increase in the lanthanum content, as also indicated by the Raman and FTIR results. Catalytic activity continuously increased with the increase in basicity, while no correlation with the catalysts’ acid properties was proved. Conversely, DMC selectivity showed a maximum as a function of the strong basic sites’ concentration, which is considered responsible for the undesired decomposition reactions not only of DMC but also of PC. The obtained results highlight that tuning the basic sites’ strength distribution is crucial for optimizing the DMC yield.

## Figures and Tables

**Table 1:** Chemical composition, structural and textural properties of the prepared oxide catalysts.

Sample	Ce/La	CeO_2_ Crystallite Size	Textural Properties		
	(mol/mol)	*D*c (nm)	*S*_BET_ (m^2^ g^−1^)	*V*_p_ (cm^3^ g^−1^)	*d*_p,max_ (nm)
CeO_2_	-	5	189	0.32	6.6
CeLa(75:25)	4.02	5	157	0.35	9.8
CeLa(50:50)	1.32	5	134	0.36	11.8
CeLa(25:75)	0.43	n.d. ^1^	117	0.37	13.6
La_2_O_3_	-	-	74	0.31	13.9

^1^ not determined.

**Table 2:** Amount of acid (*n*_A_) and basic (*n*_B_) sites (μmol m^−2^) for the synthesized oxide catalysts.

Sample	*n*_A,s_^a^	*n*_A,m_^b^	*n*_A,w_^c^	*n*_A,tot_^d^	*n*_B,s_^a^	*n*_B,m_^b^	*n*_B,w_^e^	*n*_B,tot_^f^
CeO_2_	0.07	0.16	2.03	2.26	0.05	0.30	0.37	0.72
CeLa(75:25)	0.09	0.21	2.02	2.32	0.18	0.64	0.41	1.22
CeLa(50:50)	0.11	0.13	1.78	2.01	0.29	0.99	0.64	1.92
CeLa(25:75)	0.10	0.21	2.44	2.75	0.28	0.93	0.57	1.79
La_2_O_3_	0.09	0.05	0.82	0.97	0.53	1.47	0.77	2.77

^a^ *Q*_diff_ ≥ 150 kJ mol^−1^; ^b^ 110 ≤ *Q*_diff_ < 150 kJ mol^−1^; ^c^ 60 ≤ *Q*_diff_ < 110 kJ mol^−1^; ^d^ *Q*_diff_ ≥ 60 kJ mol^−1^; ^e^ 40 ≤ *Q*_diff_ < 110 kJ mol^–1^; ^f^ *Q*_diff_ ≥ 40 kJ mol^–1^.

**Table 3:** Catalytic results for the transesterification of PC with methanol at 160 °C under autogenic pressure (15 bar). Other experimental conditions were: CH_3_OH/PC = 10 mol/mol; catalyst/PC = 0.03 g/g; reaction time = 4 h.

Catalyst	*X*_PC_	*A*_c_	*S*_DMC_	*S*_Interm_	*Y*_DMC_	DMC/PG
	(mol%)	(mmol m^−2^ h^−1^)	(mol%)	(mol%)	(mol%)	(mol/mol)
blank run	15		11	46	2	0.46
CeO_2_	58	0.25	82	6	48	0.91
CeLa(75:25)	55	0.28	84	6	46	0.91
CeLa(50:50)	60	0.36	86	6	52	0.90
CeLa(25:75)	54	0.38	87	7	47	0.90
La_2_O_3_	57	0.63	74	6	42	0.71

## Data Availability

Not applicable.

## Supplementary Materials

The following are available online at https://www.mdpi.com/article/10.3390/ma14174802/s1, Table S1: Products distribution (mol%) by GC analysis. Experimental conditions: *T* = 160 °C; autogenic pressure (15 bar); CH_3_OH/PC = 10 mol/mol; catalyst = 3 wt% (referred to the PC mass); reaction time = 4 h, Figure S1: FTIR spectra of La_2_O_3_ at RT. A: after outgassing at 450 °C (a), exposure to CO_2_ (b), and subsequent outgassing at RT (c) and 450 °C (d); B: after outgassing at 450 °C (a), exposure to CO_2_ (b), difference spectrum (e) obtained by subtracting the (a) profile by the (b) spectrum, Figure S2: TG (A) and DTG (B) curves for the prepared oxide catalysts,Table S2: Weight losses from TG analysis for the prepared oxide catalysts.
