# Supplementary material for: Synthesis of Dimethyl Carbonate by Transesterification of Propylene Carbonate with Methanol on CeO2-La2O3 Oxides Prepared by the Soft Template Method
Source: Materials (Basel). 2021 Aug 24;14(17):4802. doi: 10.3390/ma14174802 (PMC8432469; doi:10.3390/ma14174802)
Supplement: Supplementary file 1 [file materials-14-04802-s001.zip › materials-1347261-supplementary.pdf]

Supplementary Material

Table S1. Products distribution (mol %) by GC analysis. Experimental conditions: T = 160 °C; autogenic pressure (15 bar); CH_3OH/PC = 10 mol/mol; catalyst/PC = 0.03 g/g; reaction time = 4 h.

**Products**	**CeO_2**	**CeLa(75:25)**	**CeLa(50:50)**	**CeLa(25:75)**	**La_2O_3**
DME	0.6	0.4	0.4	0.5	0.3
DMC	44.7	45.4	45.2	45.1	39.8
2HPMC + 1HPMC	3.3	3.5	3.0	3.4	3.5
PO	0.4	0.1	0.2	0.2	-
1MO-2P	1.4	0.4	0.4	0.4	-
2MO-1P	0.5	0.2	0.2	0.2	0.1
PG	48.9	49.8	50.3	49.9	56.2
DPGs	0.24	0.24	0.18	0.30	0.05

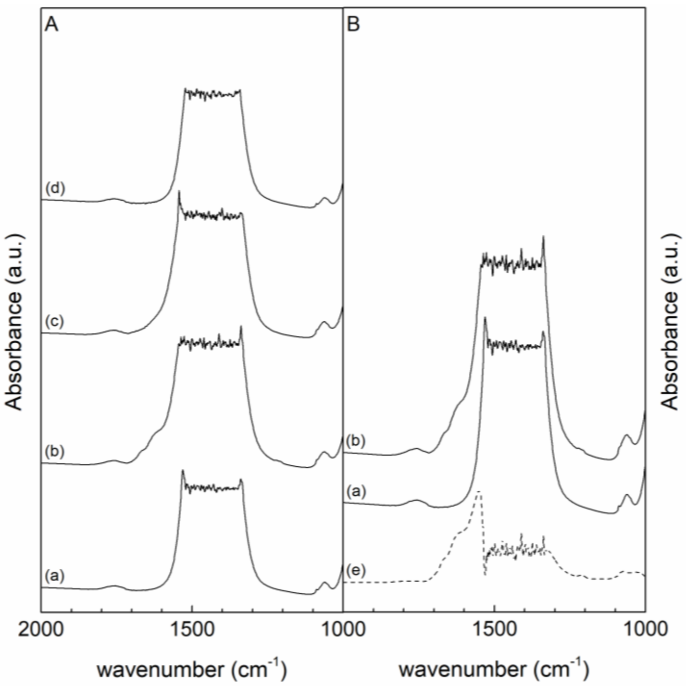

Figure S1. FTIR spectra of La_2O_3 at RT. A: after outgassing at 450 °C (a), exposure to CO_2 (b), and subsequent outgassing at RT (c) and 450 °C (d); B: after outgassing at 450 °C (a), exposure to CO_2 (b), difference spectrum (e) obtained by subtracting the (a) profile by the (b) spectrum.

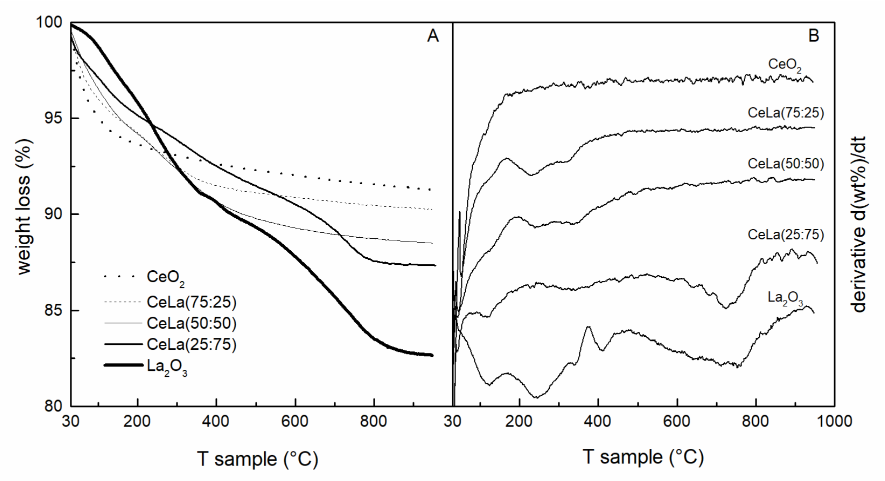

Figure S2. TG (A) and DTG (B) curves for the prepared oxide catalysts.

Table S2. Weight losses from TG analysis for the prepared oxide catalysts.

**Samples**	**Weight loss (%)**		
	**(30-200 °C)**	(200-400 °C)	(400-950 °C)
CeO_2	6.36	1.02	1.35
CeLa(75:25)	5.75	2.75	1.23
CeLa(50:50)	5.81	3.52	2.17
CeLa(25:75)	4.85	2.64	5.18
La_2O_3	4.18	5.20	7.96
